# Application of next generation sequencing in genetic counseling a case of a couple at risk of cystinosis

**DOI:** 10.1186/s12881-020-01167-y

**Published:** 2020-12-12

**Authors:** Mouna Ouhenach, Abdelali Zrhidri, Imane Cherkaoui Jaouad, Wiam Smaili, Abdelaziz Sefiani

**Affiliations:** 1 Department of Medical Genetics, National Institute of Health, 27, Avenue Ibn Battouta, BP 769, Rabat, Morocco; 2 Human Genomics Center, Faculty of Medicine and Pharmacy, Rabat, Morocco

**Keywords:** Next generation sequencing, Consanguinity, Genetic counseling, Cystinosis

## Abstract

**Background:**

In Morocco, consanguinity rate is very high; which lead to an increase in the birth prevalence of infants with autosomal recessive disorders. Previously, it was difficult to diagnose rare autosomal recessive diseases. Next Generation Sequencing (NGS) techniques have considerably improved clinical diagnostics. A genetic diagnosis showing biallelic causative mutations is the requirement for targeted carrier testing in parents, prenatal and preimplantation genetic diagnosis in further pregnancies, and also for targeted premarital testing in future couples at risk of producing affected children by a known autosomal recessive disease.

**Methods:**

In this report, we present our strategy to advise a future couple of first cousins, whose descendants would risk cystinosis; an autosomal recessive lysosomal disease caused by mutations in the *CTNS* gene. Indeed, our future husband’s sister is clinically and biochemically diagnosed with cystinosis in early childhood. First, we opted to identify the patient’s *CTNS* gene abnormality by using (NGS), then we searched for heterozygosity in the couple’s DNA, which allows us to predict the exact risk of this familial disease in the future couple’s offspring.

**Results:**

We have shown that the future husband, brother of the patient is heterozygous for the familial mutation. On the other hand, his future wife did not inherit the familial mutation. Therefore, genetic counseling was reassuring for the risk of familial cystinosis in this couple’s offspring.

**Conclusions:**

We report in this study, one of the major applications of (NGS), an effective tool to improve clinical diagnosis and to provide the possibility of targeted premarital carrier testing in couples at risk.

## Background

Morocco is one of the countries with a high rate of consanguinity in the Mediterranean, where consanguineous marriages account for 15.25% of all marriages, and 58.46% of them are between first cousins [1]. Autosomal recessive disorders are strongly associated with consanguineous marriages; the frequency of these marriages are estimated at 59.09% among Moroccan families with autosomal recessive disorders [1].

Previously, the large number of potential genes and the phenotypic variability associated with many known genetic causes, have made the diagnosis of rare autosomal recessive disorders very difficult. In recent years, significant advances are achieved using next generation sequencing (NGS) technology in medical practice especially in the diagnosis of children with autosomal recessive diseases. Indeed, NGS has significantly improved clinical diagnostic compared to traditional sequencing methods. Thus, so an accurate diagnosis will enable more appropriate genetic counseling and targeted parental carrier testing, prenatal and preimplantation genetic diagnosis in subsequent pregnancies, and targeted premarital genetic testing in at-risk couples.

Here we report our experience with targeted premarital testing in a first-degree consanguineous couple, who have a family history of a severe form of cystinosis.

Cystinosis is a rare autosomal recessive lysosomal storage disorder, described in 1903 as a familial cystine accumulation [2], leading to cellular dysfunction, it’s a systemic disease whereas the kidney involvement is the most serious clinical event, driving before the age of 20 to end-stage renal disease in more than 90% of patients [3, 4].

Although cystinosis is a monogenic disease, caused by mutations in the *CTNS* gene, a large gene of 12 exons distributed across approximately 23 kb of genomic DNA, encodes the protein cystinosin and maps to chromosome 17p13. Depending on the severity of mutations affecting the *CTNS* gene, there is three major clinical presentations of the cystinosis disease: the infantile nephropathic form (MIM: 219800), the juvenile nephropathic form (MIM: 219900), and the ocular non-nephropathic form (MIM: 219750) [4–6].

## Methods

### Patients

**Fig. 1:**
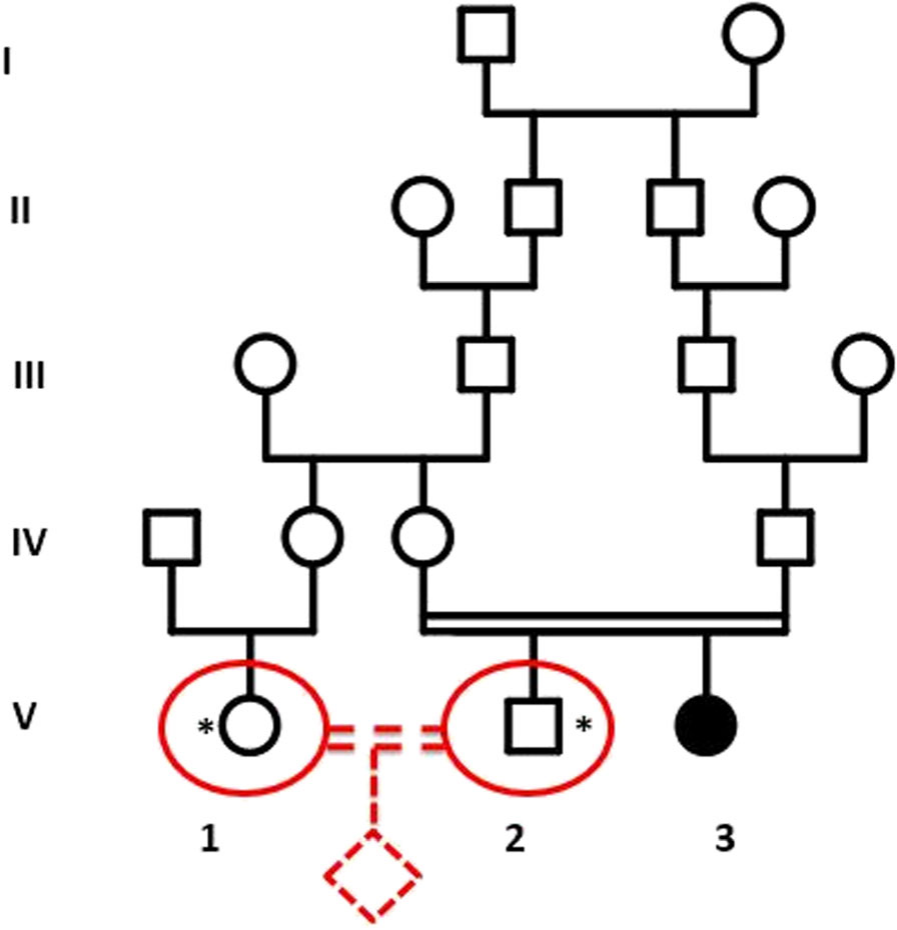
Pedigree of the studied couple (indicated with asterisks). Affected patient with cystinosis is shown by black circle (In the chapter “Patients”)

A Moroccan couple of first-degree cousins intending marrying (Individuals V-1 and V-2; Fig. 1) were referred to our specialized in genetic counseling clinic. The future husband has a sibling affected by cystinosis with an early renal failure. Therefore, the couple worried about their offspring and came to our center for genetic counseling. Accordingly, we informed the couple about the increased risk of having children with cystinosis then we proposed them more precise genetic counseling.

The patient, the future husband’s sister (patient V-3; Fig. 1), is a 22-year-old girl, diagnosed with cystinosis in early childhood. Actually, she has a severe form of cystinosis, which progressed towards renal failure at the age of 6 years old.

### Molecular studies

In a first step, we chose to identify the *CTNS* gene pathogenic mutation of cystinosis in the patient (V-3; Fig. 1). As cystinosis is a monogenic disease due to abnormalities in *CTNS* gene, which is a large gene of 12 exons, we chose to perform a target next-generation sequencing using a gene panel of hereditary diseases (Ion AmpliSeq™ Inherited Disease Panel), which include *CTNS* gene, then we confirmed the patient’s mutation. In a second step, we screened for heterozygosity of the familial mutation in our couple. Consequently, we were able to predict the exact risk of this familial disease in their offspring.

Written informed consent was obtained from the patient and the future couple prior to the implementation of the genetic study reported here.

### Detection of the familial Cystinosis causative mutation

#### -DNA and NGS

Prior to perform the genetic study reported here, written informed consent was obtained from the patient and the future couple (patient V-3 and the individuals V-1 and V-2; Fig. 1). we Used Invitrogen kit (PureLink™ Rapid Gel Extraction and PCR Purification Combination Kit/K220001) to extract genomic DNA from peripheral blood lymphocytes, and then we used the Qubit dsDNA HS (High Sensitivity) Assay Kit to quantify DNA with Qubit® Fluorometer (Invitrogen™), according to the manufacturer’s instructions.

According to the NCBI ClinVar database, the Ion AmpliSeq™ Inherited Disease Panel can provide a high degree of multiplexed target selection of all exons and some gene introns associated with more than 700 unique genetic diseases. In our case, we analyzed all coding exons of the CNTS gene. Libraries were prepared using Ion AmpliSeq Library Kit v2.0 (Life Technologies, Carlsbad, CA, USA) for this patient and for two other patients affected by other hereditary diseases. The panel contains three primer pools. For amplification, 2 μL of 5× Ion Ampliseq HiFi Mix (Thermo Fisher Scientific), 2 μL of 5× Ion Ampliseq™ inherited disease pool, 10 ng of genomic DNA per reaction, and maked up to a final volume of 10 μL with nuclease-free water. The final 10 μL polymerase chain reaction (PCR) mixture was applied with a temperature profile of 99 °C for 2 min, 99 °C for 15 s, and 60 °C for 8 min for 14 cycles, and finally kept at 10 °C. The primer sequences were partially digested. Adapters and one of 16 barcodes of the Ion Xpress Barcode Adapters 1 to 16 Kit (Thermo Fisher Scientific Life Sciences Solutions, Carlsbad, CA, USA), was applied to each sample. The library was quantified using Qubit dsDNA HS analysis kit (Molecular Probe, Eugene, OR, USA) on Qubit 2.0 fluorometer, and an equimolar amount of each library is used to prepare templates for clonal amplification. Emulsion PCR was performed on the OneTouch2 system (Life Technology in Carlsbad, California, USA) using the Ion PGM HI-Q OT2 kit (Life Technology in Carlsbad, California, USA). The template was enriched using Ion OneTouch ES (Carlsbad, California, U.S. Life Technology Company), and Ion 318 Chip v2 was prepared for loading (Carlsbad, California, U.S. Life Technology Company). According to the manufacturer’s instructions, Ion PGM HI-Q sequencing was used to perform sequencing runs on the Ion Torrent Personal Genome Machine (PGM, Life Technologies).

The Torrent Mapping Alignment Program aligner implemented in Torrent Suite software (Thermo Fisher Scientific) v5.4 was used to compare the raw sequence data in FASTQ format with the hg19 human reference genome. For SNV calling, we used plug-in Torrent Variant Caller v5.2.0.34 (Thermo Fisher Scientific) to set up a variant call format file. Then we used a default setting of germline low-stringency parameters: the minimal variant frequency is 0.1, the minimum variant quality is 10, the minimum coverage is 5 ×, the maximum strand bias is 0.98, and the minimum variant score is 10, for Torrent Variant Caller analysis. Thus, the candidate variants were only obtained when variant frequency at a given position is ≥20% and variant coverage is ≥20×. IGV (version 2.1.16) was used to view the mapping and annotation of the sequence on the graphical interface.

Reported variants are confirmed in the Human Gene Mutation Database (HGMD, http://www.hgmd.cf.ac.uk/ac/index.php), Clinvar and previous publications.

The sequence data analysis detected a potential deletion spanning exons 4 and 5 in the *CTNS* gene.

#### -RNA and RT- PCR

Initially, Total RNA was isolated from leukocytes using an RNA extraction kit (RNeasy Mini Kit, Qiagen GmbH, Hiden, Germany), and then cDNA was performed using Superscript III Reverse transcriptase (Life Technologies) according to the manufacturer’s protocol. Thus, cDNA amplification of the *CTNS* gene of the patient (V-3; Fig. 1), was performed to confirm the deletion (a potential deletion spanning exon 4 and 5), using the primers [forward: ACTGGGCGAAGGGAGGACT and reverse CACTCCAGGAGGCACCACA]. The reaction *products were* analyzed by 1% agarose gel *electrophoresis*. Then, Sanger sequencing was done using dye terminator chemistry (ABI Prism BigDye v3.1) and run on automated sequencer Applied Biosystems Prism 3130 DNA Analyzer.

### Determination of genetic profile of the couple at risk

In order to determine our couple’s (Individuals V-1, V-2; Fig. 1) genetic profile for the familial mutation, we used RT-PCR, the same method as above. Amplified products were also analyzed on a 1% agarose gel electrophoresis. Then Sanger sequencing is done using dye terminator chemical reagents (ABI Prism BigDye v3.1) and runs on the automated sequencer Applied Biosystems Prism 3130 DNA Analyzer.

## Results

### Detection of the familial Cystinosis causative mutation

**Fig. 2:**
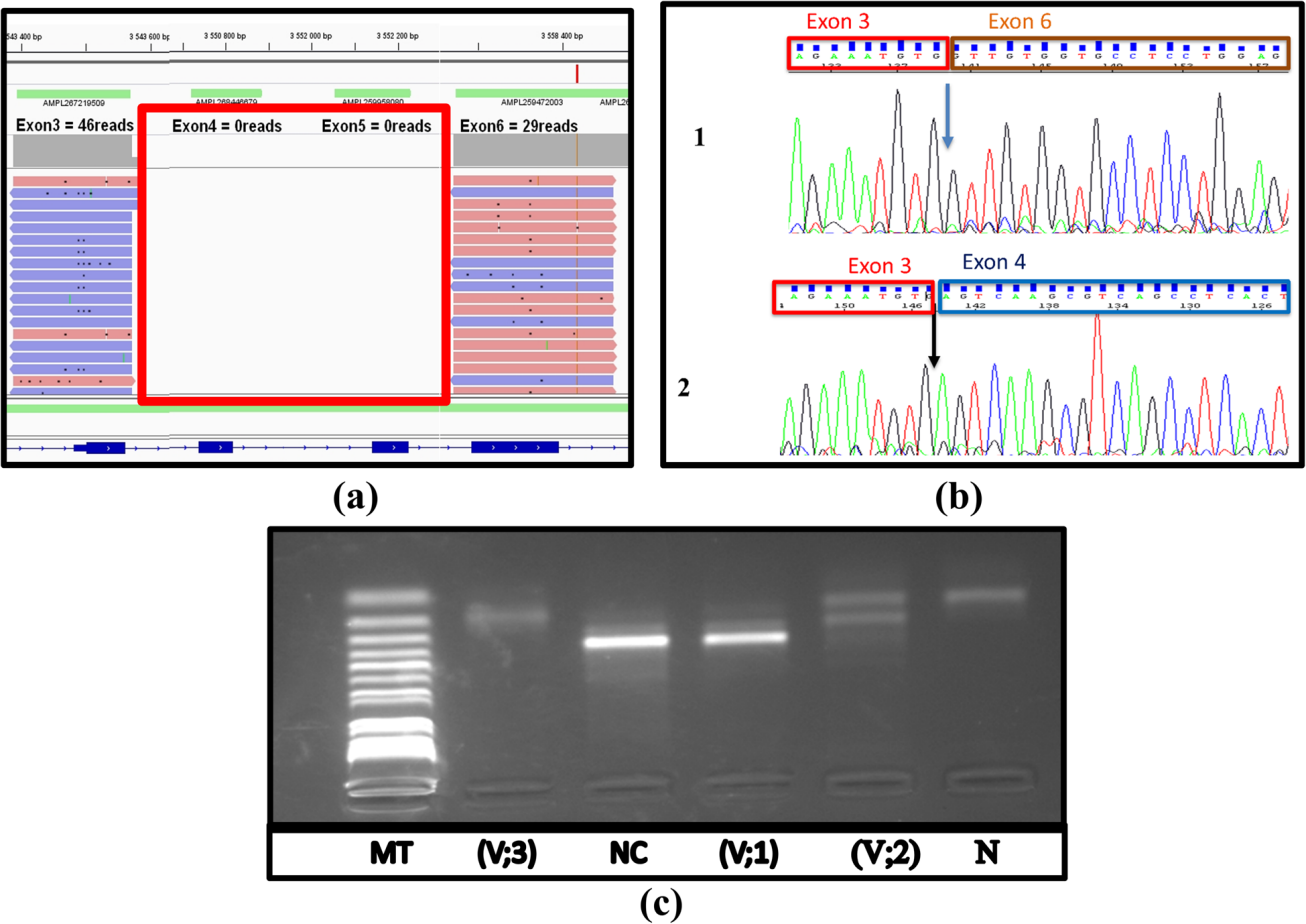
(**a**) The IGV image showing a deletion of two exons 4 and 5 in the *CTNS* gene detected by the Ion Torrent next-generation sequencing platform. (**b**) Sequencing diagram of cDNA showing the homozygous deletion of the two exons 4 and 5 in *CTNS* gene (1) in compare to an normal sequence (2). (**c**) Image of agarose gel after RT-PCR amplification products electrophoresis. (V; 3)**:** the RT-PCR amplified products gel analysis showing the presence of a small fragment comparing to those of a normal sample. (NC)**:** the RT-PCR reaction products gel analysis of a normal sample. (V; 2)**:** Agarose gel analysis of the RT-PCR amplified products of the future husband, which showed the presence of two fragments, one fragment has the same size as those of the control sample and the other fragment has the same size of the patient’s sample. (V; 1)**:** The Agarose gel analysis of the future wife, showing the presence of one fragment of the amplified target cDNA who have the same size as those of the control sample. (N): Negative control. (In the chapter “Results”)

To confirm the diagnosis of cystinosis at the molecular level, we carried out a mutational analysis as described in the patients and methods section. As follows, we used targeted next generation sequencing to detect mutations in the promoter and exons of the *CTNS* gene in our cystinosis affected family member (patient V-3; Fig. 1). The sequence data analysis detected a potential deletion spanning exons 4 and 5 (no reads are generated for these tow exons (Fig. 2a)) and no other pathogenic variant is detected in *CTNS* gene. Then, we performed RT-PCR and cDNA Sanger sequencing, which confirmed the NGS suspected deletion (Fig. 2b). This result allows us to identify the causal mutation of cystinosis in this family: homozygous deletions carrying both exons 4 and 5 [c.(61 + 1_62–1)_(225 + 1_226–1); NM_004937.3] of the *CTNS* gene, according to the HGVS format (Fig. 2a).

### Determination of genetic profile of the couple at risk

In order to determine our couple’s genetic profile, we used the RT-PCR in three individuals (the couple and the affected patient (Fig. 1: Individuals V-1, V-2, and the patient)). The agarose gel analysis of the patient’s RT-PCR reaction product (V-3; Fig. 2c) shows that the amplified cDNA fragments are smaller than those of the control sample. We compared this result to our couples RT-PCR products: The future husband’s agarose gel analysis of the RT-PCR reaction products showed the presence of two fragments (V-2; Fig. 2c), one fragment has the same size as those of the control sample, and the other fragment has the same size as the amplified fragment of the affected patient. Therefore, the future husband has two alleles, one allele carrying the deletion, and one normal allele, so he is heterozygous for the familial mutation of cystinosis. Regarding the future wife (V-1; Fig. 2c), agarose gel analysis revealed the presence of one amplified target cDNA fragment at the same size as those of the control sample (V-1; Fig. 2c). Accordingly, the future wife is homozygous normal, so she is not a carrier of the familial mutation.

### The genetic counseling

Therefore, genetic counseling is reassuring for the risk of cystinosis due to the familial mutation in their offspring. However, the couple remains at risk due to inbreeding.

## Discussion

We are describing a family with an autosomal recessive disease called cystinosis, and we presented our strategy to advise a consanguineous future couple of first-degree cousins (Individuals V-1 and V-2; Fig. 1), whose descendants would be at risk of this disease. The awareness of the risk of consanguineous marriages is getting higher in our population; we receive in our Department of Medical Genetics, National Institute of Health, Rabat, Morocco, many cases of genetic counseling of consanguineous families and couples concerned about their descendants. The aim of this study is to report one the major application of the Next-generation sequencing (NGS) technology in genetic counseling.

This couple (Individuals V-1 and V-2; Fig. 1) that we received in our consultation, were worried about their offspring, they are a first-degree consanguineous couple, who have a family member affected with severe infantile cystinosis (MIM: 219800). Our genetic counseling strategy is to perform an accurate molecular diagnosis of cystinosis disease in this family and then determine the genetic profile of the familial mutation in our couple.

Cystinosis disease is a rare lysosomal storage disease; it is a systemic disease whereas kidney involvement is the most serious clinical event. The prevalence of cystinosis disease is known in some populations, it varies between 1/192,000 in Australia to 1/26,000 in France [7, 8]. However, the incidence of cystinosis disease is still lacking in the North African population.

Cystinosis disease is due to biallelic mutations in the *CTNS* gene (17p13.2), which is a gene of 12 exons cystinosin. A large number of mutations are found in *CTNS* gene, about 168 reported in the Human Gene Mutation Database at the Institute of Medical Genetics in Cardiff (HGMD, http://www.hgmd.cf.ac.uk/ac/gene.php?gene=CTNS). The most common pathogenic mutation is a 57-kb deletion, which represent nearly 50% of *CTNS* mutant alleles in patients of North European and North American origin [9, 10], but no recurrent mutation has been found in the north African or middle eastern population.

In our case, due to the large large number of *CTNS* gene exons, we used the next generation sequencing to carry out a molecular diagnosis of cystinosis disease in this family. Furthermore, cystinosis disease is due to biallelic pathogenic mutation, so even a large deletion can also be suspect by this technology.

**Fig. 3:**
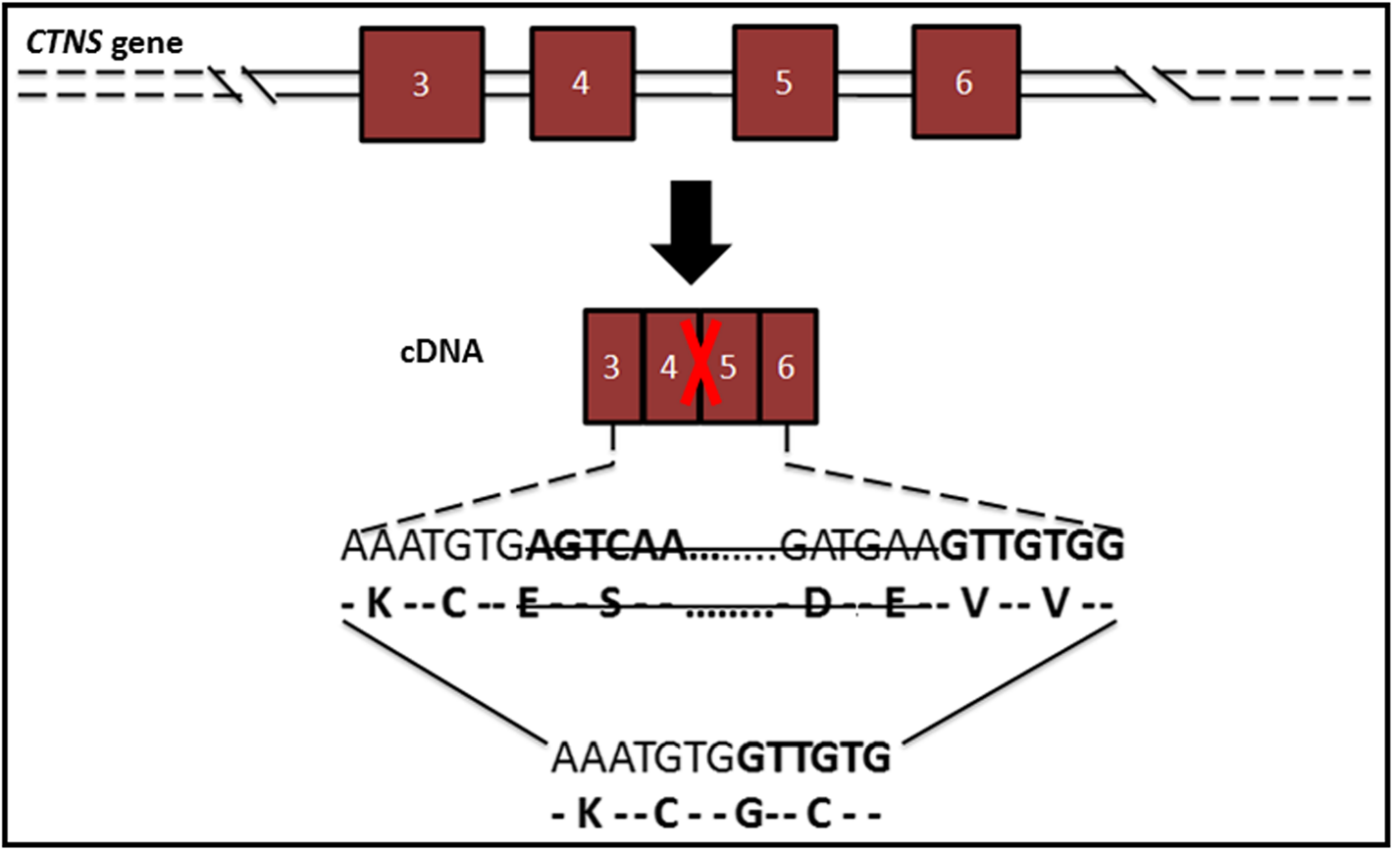
Schematic representation of the Exon 4 and 5 deletion of *CTNS* gene and their consequence in cDNA of this region of the gene. (In the chapter “Discussion”)

Effectively, the analysis of our patient (V-3; Fig. 1), identified a deletion spanning exons 4 and 5 of *CTNS* gene ((a); Fig. 2). We confirmed this mutation [c.(61 + 1_62–1)_(225 + 1_226–1), NM_004937.3] by RT-PCR ((c); Fig. 2) and cDNA Sanger sequencing ((b); Fig. 2). This deletion leads to a short mRNA, resulting in a frameshift and truncated version of CTNS protein (Fig. 3), and was already reported as pathogenic [11]. That explains the childhood-onset and the severe form of our patient’s phenotype. Therefore, we conclude that this allele variant should be classified as pathogenic and there is no need to screen it in a healthy control.

Next generation sequencing technology has helped us to obtain a more rapidly, and more accurate diagnosis. NGS is potentially allowing for more efficient genetic counseling, premarital targeted testing in the couple at risk, and did helped us to predict the exact risk of this familial disease in this couple’s offspring; therefore, we were able in this case to reassure the couple about the risk of cystinosis in their descendent. However, the couple remains at risk for other recessive diseases due to the inbreeding.

## Conclusions

Here we reported one of the major applications of next generation sequencing. These tests become more accessible and faster, it would be more cost-benefit for patients and their families. Our perspective is to create a screening test for the most common autosomal recessive disease in our population, as a health public strategy in order to reduce the prevalence of these diseases.

## Data Availability

Web links and the full names of the data banks/repositories corresponding to all of the datasets obtained from web-based sources and subsequently analysed in this study are cited below: -human genome 19 reference: https://www.ncbi.nlm.nih.gov/assembly/GCF_000001405.13/ - NM_004937.3: http://www.ncbi.nlm.nih.gov/nuccore/NM_004937.3 - Human Gene Mutation Database: http://www.hgmd.cf.ac.uk/ac/index.php - List of inherited diseases associated to the Ion AmpliSeq™ Inherited Disease Panel target gene, according to NCBI ClinVar database, wich include cystinosis disease: ftp://ftp.ncbi.nlm.nih.gov/pub/clinvar/gene_condition_source_id The principal data (causative variant in the patient and, agarose gel analysis of the RT-PCR amplification products of the patient and the couple and Sequencing diagrams of cDNA) generated and/or analyzed during the current study are included in the published article. The complete datasets used and/or analyzed during the current study are available from the corresponding author upon request.

## Abbreviations

NGS: Next Generation Sequencing
PGM: Personnel Genome Machine
DNA: Deoxyribonucleic acid
PCR: Polymerase chain reaction
cDNA: Complementary Deoxyribonucleic Acid
RT-PCR: *Reverse transcription polymerase chain reaction*
